# Ultrasonographic length of morphologically-normal kidneys in children presented to a premier tertiary healthcare setting of Sri Lanka

**DOI:** 10.1186/s12882-019-1377-z

**Published:** 2019-05-22

**Authors:** W. D. Duminda, K. G. Pathirana, M. U. J. Fernando, R. A. N. K. K. Samarasinghe, W. D. H. A. Ananda, K. S. P. Silva, C. K. Dissanayake, P. K. B. Mahesh

**Affiliations:** 1 Post Graduate Institute of Medicine, Colombo, Sri Lanka; 2 Lady Ridgeway Hospital for Children, Colombo, Sri Lanka; 3 Melbourne School of Population and Global Health, University of Melbourne, Melbourne, Australia; 4 Ministry of Health, Colombo, Sri Lanka

**Keywords:** Renal length, Predictive models of renal length, Renal length references, Sri Lanka, Ultrasonographic renal length

## Abstract

**Background:**

Accurate prediction of reference ranges of renal lengths facilitates clinical decision making. Currently a single renal-length-reference chart is used for both kidneys, which is solely based on the age of the child without adjusting for anthropometrics.

Objective of the study is to assess the length of morphologically-normal kidneys ultrasonically and to build models to predict the renal lengths of children presenting at the Radiology Department of Lady Ridgeway Hospital for Children.

**Methods:**

A descriptive cross sectional study was done among 424 children with 233 males and 191 females at the study setting. Study population included children undergoing abdominal ultrasound scans for indications not related to renal disease. Children with a family history of renal diseases or with morphologically-abnormal kidneys were excluded. Bipolar-lengths of kidneys, gender and anthropometrics were documented. Having tested for assumptions, Wilcoxon-signed rank test, Mann-Whitney U test and multiple linear regression were used.

**Results:**

The mean (SD) bipor-length of right and left kidneys were 6.83 (1.43) and 7.05 (1.36) respectively (*p* < 0.001). Age, height and weight were significantly correlated with the renal lengths (*p* < 0.05). Until 16 months, there was a significant difference between the renal lengths between males and females (*P* < 0.05). Yet the association with gender was not significant from 17 months and in overall. Until 16 months, the best linear-regression equation (*p* < 0.001) for the left kidney was; 3.827 +  0.019(length in centimeters) +  0.141(weight in kilograms) - 0.023(age in months) - 0.347(for male sex). For the right kidney, it was; 3.888 + 0.020(length or height) + 0.121(weight) - 0.037(age) - 0.372 (for male sex). The respective R squares were 59.2 and 53.5% with VIF (Variance-Inflation-Factor) ranging from 1.06 to 2.08. From 17 months, best equation for left kidney (p < 0.001) was; 5.651+ 0.022(age) + 0.01(BMI). For right kidney it was; 5.336 + 0.022(age) + 0.012(BMI). The R squares were 62.5 and 66.1% with VIF being 1.

**Conclusions:**

The established models explain more variability for children above 17 months. Both renal lengths are affected significant by the body’s’ anthropometric parameters. For each kidney, separate normograms of renal lengths which are local-context-specific must be prepared. Further research must be promoted.

**Electronic supplementary material:**

The online version of this article (10.1186/s12882-019-1377-z) contains supplementary material, which is available to authorized users.

## Background

Renal dimensions assessed by imaging techniques like ultrasonography would facilitate clinical decision making in children. In order to facilitate this, determination of normal renal ranges is essential. Once these normal ranges are determined, they function as a baseline tool for early interventions [1]. Though the renal volume is the best dimension of the kidney, renal length is regarded as the most useful parameter. This is due to the less-complexity of measurements and the lower inter-observer variability. It enables distinguishing acute from chronic renal diseases and enables the detection of renal-hypoplasia [2].

Bi-phasic patterns of the rate of growth of renal-length have been noted in global literature [2, 3]. Hence in many manuscripts two regression models have been mentioned for different age groups [3]. The left kidney has been described as having a greater longitudinal length than the right [4–7]. Hence in the literature different regression models have been proposed for left and right kidneys [1, 2, 4]. In some instances, separate models have been developed for length predictions when different radiological modalities are used [8].

The correlations of the anthropometric body indices versus dimensions of several body organs have been documented in litearture [9, 10]. It is well known that renal size is related to age, height and weight of children. Many studies have shown that height correlates best with renal length [4, 11–13]. Correlation of renal length with the body mass index (BMI) is also observed in some studies in other countries [4, 13, 14]. Globally the association of gender with the renal length has shown diverse findings. In an Indian study, though the renal sizes in children correlated with body length and body surface area, there was no significant difference between boys and girls [15]. However a study done in Copenhagen revealed a difference in renal dimensions between males and females [16].

The Lady Ridgeway Hospital for Children (LRH) is the premier tertiary care children’s hospital in Sri Lanka providing care to the children admitted from all over the country. The Department of Radiology and Diagnostic Imaging of the LRH plays the major role in diagnostic and some therapeutic procedures in children with kidney-related diseases. In the LRH, a single renal-length-reference chart is currently used for lengths of both kidneys, which does not provide adjusted parameters for anthropometrics. Therefore development of local-context-specific separate normograms of renal lengths would mark an advancement of the quality of healthcare.

Aims of the study were to assess the length of morphologically normal kidneys ultrasonically and to build models to predict the renal lengths with the view of preparing prospective context-specific normograms.

## Methods

A descriptive cross sectional study was done at the Department of Radiology, the Lady Ridgeway Hospital for Children (LRH) Colombo, Sri Lanka from October 2016 to December 2017. Study population included patients up to completion of 16 years who underwent ultrasound scans abdomen for indications not related to renal disease were selected for the study. Patients with past history or family history of renal disease and patients with morphologically abnormal kidneys on ultrasound scans were excluded.

Sample size calculation was done with the formula for the estimation of a quantitative variable in a cross sectional study. With a significant level as 5%, the needed sample size at data analysis stage was 315 [7, 17]. Taking into consideration a non-response rate of 25%, 420 children were needed to be recruited at the data collection stage. Altogether 424 children were recruited. The study instruments included a pre-tested interviewer administered questionnaire and a data extraction form. Ultrasound scans were done by one of the investigator in all recruited children. The ultrasound scanner was “Toshiba Aplio 500”. A sample image with measurements has been included in the supplementary materials. (See Additional file 1: Supplementary Material-1 and Additional file 2: Supplementary Material-2). Morphologically normal kidneys were identified after ultrasound scanning and got the maximum bipolar length of each kidney in coronal plane. The renal lengths were documented after repeating measurement for three times in the supine position and by getting the maximum value.

Data were entered in to a pre-designed sheet in Statistical Package for Software Sciences (SPSS version 17). Descriptive statistics were used in describing the data. Normality testing of variables were done with graphical techniques and with Kolmogorov- Smirnov test. The distributions were found to be non-normal (See Additional file 3: Supplementary Material-3). The difference between the left and right kidneys were evaluated by Wilcoxon signed rank test. The associations of the lengths with the categorical variables were evaluated with Mann Whitney U test and the numerical variables with the Spearman correlation coefficient. After analysis for the fulfillment of assumptions, multivariant analysis was done with multiple linear regression. Model building was done with purposive selection. Selection of the best method was done by considering R square values and variance inflation factors (VIF).

The informed written consent was taken from the parent/guardian. Ethical approval was obtained from the Ethics Committee of the Lady Ridgeway Hospital for Children, Sri Lanka.

## Results

During the data collection period 424 participants were recruited with 233 (55%) males and 191 (45%) females. The distribution of age stratified by the gender is shown in Table 1. The mean (SD) height and weight (SD) of the study population were respectively 93.2 (33.9) cm and 19.1 (14.3) kg. The commonest indication for getting the abdominal ultrasound was abdominal pain (*N* = 144, 38%) out of which in 38 children, intussusception was suspected.

**Table 1:** Details of the participants

	Male N (%)	Female N (%)	Total N (%)
Up to 2 years	95 (59.0)	66 (41)	161 (100)
3–5 years	38 (48.7)	40 (51.3)	78 (100)
6–10 years	66 (53.6)	57 (46.4)	123 (100)
> 10 years	34 (54.8)	28 (45.2)	62 (100)

The descriptive statistics of the renal lengths are mentioned in Table 2. The left kidney was found to be longer than the right (*p* < 0.001). The difference between left and right kidneys was not constant. The median (IQR) of the difference was 0.2 (0.1 to 0.4) cm. There was no constant distribution of the differences across different ages as well.

**Table 2:** Distribution of kidney measurements in centimeters

	Right kidney	Left kidney	Association^a^
Mean (SD)	6.83 (1.43) cm	7.05 (1.46) cm	*P* < 0.001
Median (IQR)	6.6 (5.6 to 7.9) cm	6.9 (6.0 to 8.0) cm	

^a^Wilcoxon signed rank test

**Fig. 1:**
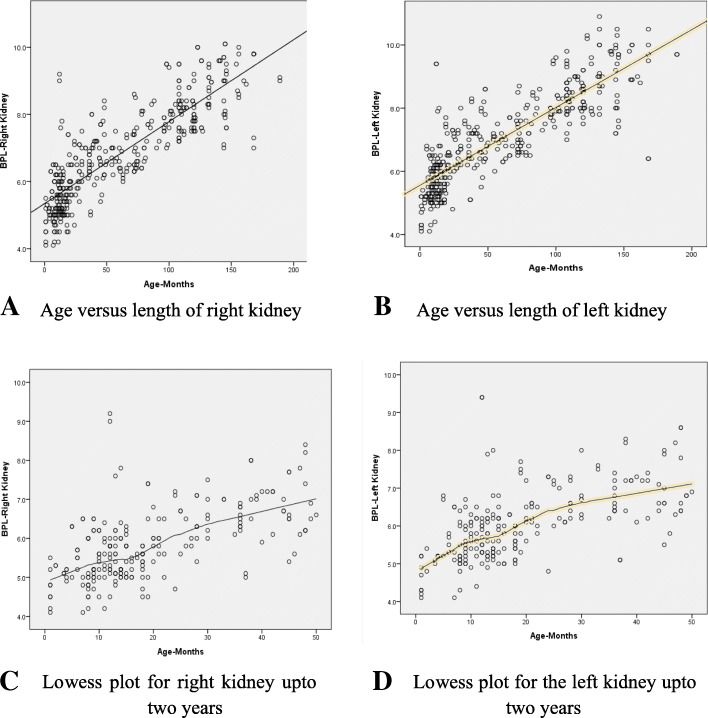
**a** to **d**: Scatter plot diagrams between renal length and age.

Figure 1a and b demonstrate the graphical representation of the association between age and the renal length. In both kidneys, the different models including linear, quadratic and cubic were explored. The linear model was found to be more suitable when analyzed with the “change of R square”. When analyzed further using the “Lowess fit-line”, the slope of increase was found to be more within the first 16 months compared to “17 months and above” (Figs. 1c and d).

The associations of the lengths of kidneys with anthropometric measurements have been summarized in Table 3. In overall the association of gender was not significant for right (*p* = 0.075) and left (*p* = 0.069) kidneys. Yet when the age groups were stratified into two categories by exploring the slope of the curve, “16 or below” category showed a significant associations (*p* < 0.05) whereas in “17 or more” category, the associations were not significant (*p* = 0.463 and *p* = 0.437).

**Table 3:** Associations of the kidneys

	Right kidney	Left kidney
Gender		
Overall	*p* = 0.075	*p* = 0.069
< 16 months	*p* < 0.001	*p* < 0.001
> 17 months	*p* = 0.463	*p* = 0.437
Age		
Overall	r = 0.861 (p < 0.001)	r = 0.858 (*p* < 0.001)
< 16 months	r = 0.262 (*p* = 0.003)	r = 0.354 (*p* < 0.001)
> 17 months	r = 0.819 (p < 0.001)	r = 0.795 (*p* < 0.001)
Height		
Overall	r = 0.847 (p < 0.001)	r = 0.847 (*p* < 0.001)
< 16 months	r = 0.391 (p < 0.001)	r = 0.451 (*p* < 0.001)
> 17 months	r = 0.744 (p < 0.001)	r = 0.736 (*p* < 0.001)
Weight		
Overall	r = 0.872 (*p* < 0.001)	r = 0.870 (*p* < 0.001)
< 16 months	r = 0.264 (*p* = 0.003)	r = 0.389 (*p* < 0.001)
> 17 months	r = 0.815 (*p* < 0.001)	r = 0.791 (*p* < 0.001)
BMI		
Overall	r = − 0.226 (*p* < 0.001)	r = − 0.234 (*p* < 0.001)
< 16 months	r = − 0.131 (*p* = 0.144)	r = − 0.062 (*p* = 0.488)
> 17 months	r = 0.079 (*p* = 0.173)	r = 0.048 (*p* = 0.405)

Age, height and weight were significantly associated with the renal lengths (p < 0.05). All associations showed positive correlations. In overall and in the category “16 or less” the BMI showed a relatively weak negatve correlation which were respectively signifiant (in the overall sample) and not significant (in the latter category). In “17 or more” category it showed a positive correlation though being weak and not statistically significant (Table 3).

Tables 4 and 5 show the multivariant analyis including the selected model diagnostics for the kidney lengths. The R square value was highest (53.5 and 59.2% for right and left kidneys) when the model included age, weight and legth for the category “16 or less”. The highest VIF value was around 2 for the two models. For the category “17 or more”, though the model with highest R square included age, length/height and weight, the highest VIF value was closer to 5. Hence the best models included age and BMI, which provided R square values of 66.1 and 62.5% for the right and left kidneys respectively.

**Table 4:** Regression analysis of the renal lengths of right kidney

	Models	Predictors	R square	Significance	VIF value/s
1–16 months	1	Age	7.1%	*P* = 0.002	1.000
	2	Age BMI	9.4%	*P* = 0.002	1.011
	3	Age Sex	16.9%	*P* < 0.001	1.031
	3	Age Length	38.3%	*P* < 0.001	1.266
	4	Age Length Weight	48.5%	*P* < 0.001	1.478 to 2.080
	5	Age Length Weight Sex	53.5%	*P* < 0.001	1.063 to 2.080
17 months and above	1	Age	65.2%	*P* < 0.001	1.000
	2	Age Length or Height	65.8%	*P* < 0.001	3.989
	3	Age BMI	66.1%	*P* < 0.001	1.000
	4	Age Length or Height Weight	69.6%	*P* < 0.001	2.722 to 4.964

**Table 5:** Regression analysis of the renal lengths of left kidney

	Models	Predictors	R square	Significance	VIF value/s
1–16 months	1	Age	12.4%	*P* = 0.002	1.000
	2	Age BMI	13.6%	*P* < 0.001	1.011
	3	Age Sex	20.7%	*P* < 0.001	1.031
	4	Age Length	42.5%	*P* < 0.001	1.266
	5	Age Length Weight	55.1%	*P* < 0.001	1.478 to 2.080
	6	Age Length Weight Sex	59.2%	*P* < 0.001	1.063 to 2.080
17 months and above	1	Age	61.9%	*P* < 0.001	1.000
	2	Age Length or Height	62.7%	*P* < 0.001	3.989
	3	Age BMI	62.5%	*P* < 0.001	1.000
	4	Age Length or Height Weight	66.0%	*P* < 0.001	2.722 to 4.964

Until 16 months, the best linear-regression equation (p < 0.001) for the left kidney was; 3.827 + 0.019(length in centimeters) + 0.141(weight in kilograms) - 0.023(age in months) - 0.347(for male sex). For the right kidney, it was; 3.888 + 0.020(length or height) + 0.121(weight) - 0.037(age) - 0.372 (for male sex). From 17 months, best equation for left kidney (*p* < 0.001) was; 5.651+ 0.022(age) + 0.01(BMI). For right kidney it was; 5.336 + 0.022(age) + 0.012(BMI).

## Discussion

This study highlighted the importance of having separate renal-length normograms for the left and right kidneys. Furthermore this emphasized the importance of considering the anthropometric measurements in clinical decision making on the renal length. Additionally this study has shown that the rate of renal growth is more in the first 16 months and that the factors affecting the renal length would be different in this period compared to 17 months or more. The hypothesis generation done by this study has opened a potential pathway for further research which would lead to the development of Sri Lanka-specific renal normograms.

The applicability of the findings of the study becomes more evident when the disease burden of the childhood urinary tract related conditions are concerned. As an example most of the patients with urinary tract related diseases presenting to the Department of Radiology of the LRH give a history of urinary tract infection (UTI) [18]. UTIs can potentially involve the renal parenchyma leading to cortical scarring affecting the renal functions. Timely detection of these children would be facilitated by context specific normograms of renal lengths.

The measurement values of the renal length may be varying with patient position and imaging plane during the scan. As mentioned in literature, the coronal and sagittal imaging planes demonstrate largest renal length measurements while imaging with prone position demonstrated smallest measurements [19]. In the present study coronal imaging plane was used enabling larger parameters.

In our research population the left kidney was found to be longer than the right. Previous studies done in other parts of the world have shown similar results [5–7]. This fact points toward the necessity of developing separate renal-reference charts for the left and right sides. Except for the size measurements, the associated factors in overall, “16 or less” category and the “17 or more” categories were found to be similar (Tables 3-5). Due to this, the possible biological plausibility of associations does not become an uncertainty in explaining the findings.

In the present study, the slope of increase in the “Lowess fit-line” is more within the first 16 months of age compared to the age 17 months and above. Similar findings have been observed in documented global literature. In a study done by Mesrobian et al (1998), it was described that the renal growth in the first seven months of age is rapid [20]. Lee et al (2014) and Cho et al. (2015) have documented that predicting the renal length of infants is relatively complicated compared to older children [3, 8]. The impact of the potential variables may not be uniform in the bi-phasic growth of kidney lengths. Hence the authors decided not to be limited to the statistical accuracy, but to consider other phenomena like biological plausibility in deciding to develop two models instead of one.

There has been no global consensus on the variables to be included in the models as well as on the demarcation of age groups for which the models are valid. Hence different models have been proposed. R-squared values range from lower levels like 20–30% up to relatively higher levels like 70–85% [3]. Characteristics of the variables affecting the renal lengths may potentially become different from setting to setting as well as from time to time. As an example with the setting specific and time specific changes of the childhood obesity, the anthropometric parameters are affected [4]. Hence a model which is developed context-specifically is more valid than another, which has been developed at another setting or time. In this regard, the present study has uncovered invaluable evidence from a lower middle income setting and has achieved the objective of development of context-specific predictive models. The way forward includes refining of these predictive models as well as external validation of these in another sample.

There were several limitations of the study. Other potential dependent variables like the volume of kidney were not included in the analysis. These additional parameters must be concerned in the prospective studies which must be encouraged. Secondly the R-squared values are still relatively low in the present study especially in the “16 or less category”. Hence the proposed models of this study could not be regarded simply as far better than other models currently available. Evaluations with external validation are essential in this regard. Further research must evaluate the influence of other potential independent factors like birth weight, body surface area which have been not included in the present study. Attempts must be done in exploring currently undetermined predictors as well.

## Conclusions

The lengths of the left and right kidneys are different. Both renal lengths are affected significant by the body’s’ anthropometric parameters. The percentage of variability explained by the models were higher for “17 months and above children” than “16 and below” category. Separate normograms of renal lengths which are local-context-specific must be prepared. Further research must be promoted to establish more robust models utilizing this evidence as an eye-opener.

## Additional files

Additional file 1: Supplementary Material 1: Sample image of left kidney. Sample image of left kidney with measurement of renal length. (JPG 36 kb)

Additional file 2: Supplementary Material 2: Sample image of right kidney. Sample image of right kidney with measurement of renal length. (JPG 41 kb)

Additional file 3: Supplementary Material 3: Normality testing of the variables. Normality test in findings of numerical variables. (DOCX 13 kb)

## Abbreviations

BMI: Body Mass Index
IQR: Interquartile Range
LRH: Lady Ridgeway Hospital for Children
SD: Standard Deviation
SPSS: Statistical Package for Software Science
UTI: Urinary Tract infection
VIF: Variance Inflation Factor
