# Supplementary figures and images for: Ultrasonographic length of morphologically-normal kidneys in children presented to a premier tertiary healthcare setting of Sri Lanka
Source: BMC Nephrol. 2019 May 22;20:183. doi: 10.1186/s12882-019-1377-z (PMC6532158; doi:10.1186/s12882-019-1377-z)

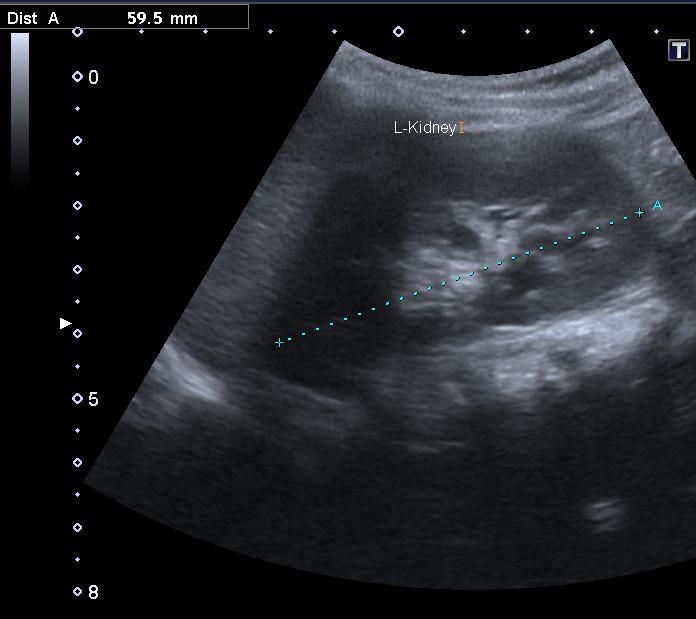

Supplement: Supplementary file 1 — Supplementary Material 1: Sample image of left kidney. Sample image of left kidney with measurement of renal length. (JPG 36 kb) [file 12882_2019_1377_MOESM1_ESM.jpg]

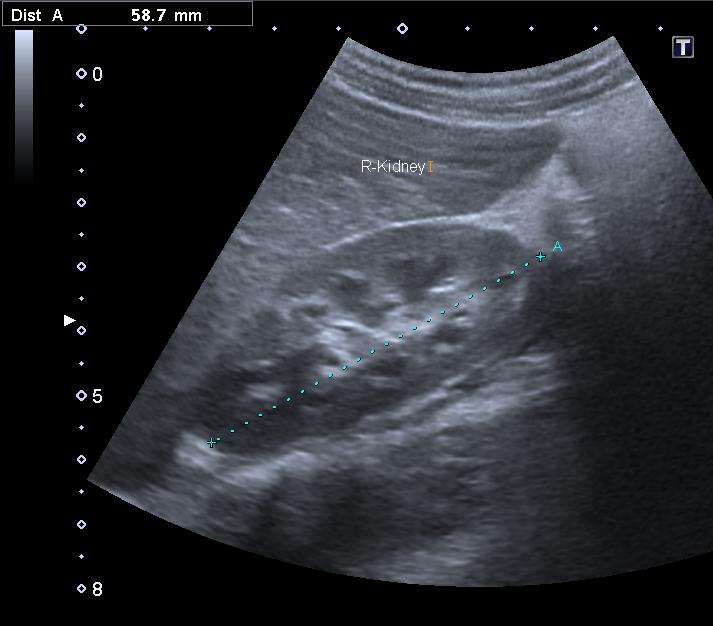

Supplement: Supplementary file 2 — Supplementary Material 2: Sample image of right kidney. Sample image of right kidney with measurement of renal length. (JPG 41 kb) [file 12882_2019_1377_MOESM2_ESM.jpg]
