# Supplementary material for: Ultrasonographic length of morphologically-normal kidneys in children presented to a premier tertiary healthcare setting of Sri Lanka
Source: BMC Nephrol. 2019 May 22;20:183. doi: 10.1186/s12882-019-1377-z (PMC6532158; doi:10.1186/s12882-019-1377-z)
Supplement: Supplementary file 3 — Supplementary Material 3: Normality testing of the variables. Normality test in findings of numerical variables. (DOCX 13 kb) [file 12882_2019_1377_MOESM3_ESM.docx]

**Supplementary Material 3.**

**Normality testing of the variables**

| **Tests of Normality** | | | | | | | |
| --- | --- | --- | --- | --- | --- | --- | --- |
| Age cut off | | Kolmogorov-Smirnov^a^ | | | Shapiro-Wilk | | |
|  |  | Statistic | df | Sig. | Statistic | df | Sig. |
| 1.00 | Age-Months | .125 | 126 | .000 | .939 | 126 | .000 |
|  | Height (cm) | .164 | 126 | .000 | .891 | 126 | .000 |
|  | Weight (kg) | .145 | 126 | .000 | .817 | 126 | .000 |
|  | BPL-Right Kidney | .149 | 126 | .000 | .860 | 126 | .000 |
|  | BPL-Left Kidney | .118 | 126 | .000 | .867 | 126 | .000 |
| 2.00 | Age-Months | .107 | 298 | .000 | .945 | 298 | .000 |
|  | Height (cm) | .071 | 298 | .001 | .975 | 298 | .000 |
|  | Weight (kg) | .174 | 298 | .000 | .855 | 298 | .000 |
|  | BPL-Right Kidney | .052 | 298 | .048 | .989 | 298 | .019 |
|  | BPL-Left Kidney | .066 | 298 | .004 | .988 | 298 | .018 |
| a. Lilliefors Significance Correction | | | | | | | |
